# Current concepts review. Management of proximal tibial fractures

**DOI:** 10.3389/fsurg.2023.1138274

**Published:** 2023-03-23

**Authors:** Patrick Gahr, Sebastian Kopf, Stephan Pauly

**Affiliations:** ^1^ Department of Trauma, Hand and Reconstructive Surgery, Rostock University Medical Center, Rostock, Germany; ^2^ Center for Orthopedics and Traumatology, Medical School Theodor Fontane, Brandenburg an der Havel, Germany; ^3^ Department of Orthopedic and Trauma Surgery, Vivantes Auguste-Viktoria-Klinikum, Berlin, Germany

**Keywords:** tibial fracture, classification, fracture fixation, minimally invasive, arthroscopic

## Abstract

The management of proximal tibial fractures has evolved significantly in recent years. While the main goals of treatment – stability, restoration of the mechanical axis, and smooth articular surfaces – remain the same, methods have advanced substantially. In diagnostics, technical progress in CT and MR imaging has led to a better three-dimensional understanding of the injury. Newly developed classification systems such as the three-column concept of Luo et al. and the 10-segment concept of Krause et al. take this into account. Accordingly, there is a trend towards tailored approaches for particular fracture localizations. Parallel to this development, there is increasing evidence of the advantages of arthroscopically assisted surgical procedures. This Current Concepts article reviews classifications, diagnostics, treatment options as well as complications in fractures of the proximal tibia.

## 1. Introduction

Proximal tibial fractures account for approximately 1% of all fractures (1). The annual incidence is estimated to be 10 per 1,00,000 inhabitants. While most proximal tibial fractures occur in men, fractures in the elderly are more common in women. In both genders, most proximal tibial fractures occur between the ages of 40 and 60 (2).

While high-energy traumata such as motor vehicle accidents cause the majority of proximal tibial fractures in men, most fractures in women are caused by low-energy mechanisms of injury such as falls during walking or cycling (2). Low-energy injuries typically cause unilateral depression-type fractures, whereas high-energy injuries can lead to comminuted fractures with significant osseous, soft-tissue, and neurovascular injuries (3). Fractures involving the tibial head can result from multidirectional forces (medial, lateral, or axial). Forces directed medially (valgus force moment) are often classic “bumper fractures” (motor vehicle vs. pedestrian accidents) (4). More complex mechanisms involve combinations of two, axial as well as varus or valgus, forces. In most cases, both shearing and compressive forces are applied to the underlying tibial plateau *via* the femoral condyle (either medially or laterally) (5).

## 2. Management of proximal tibial fractures

### 2.1. Classifications

**Figure 1:**
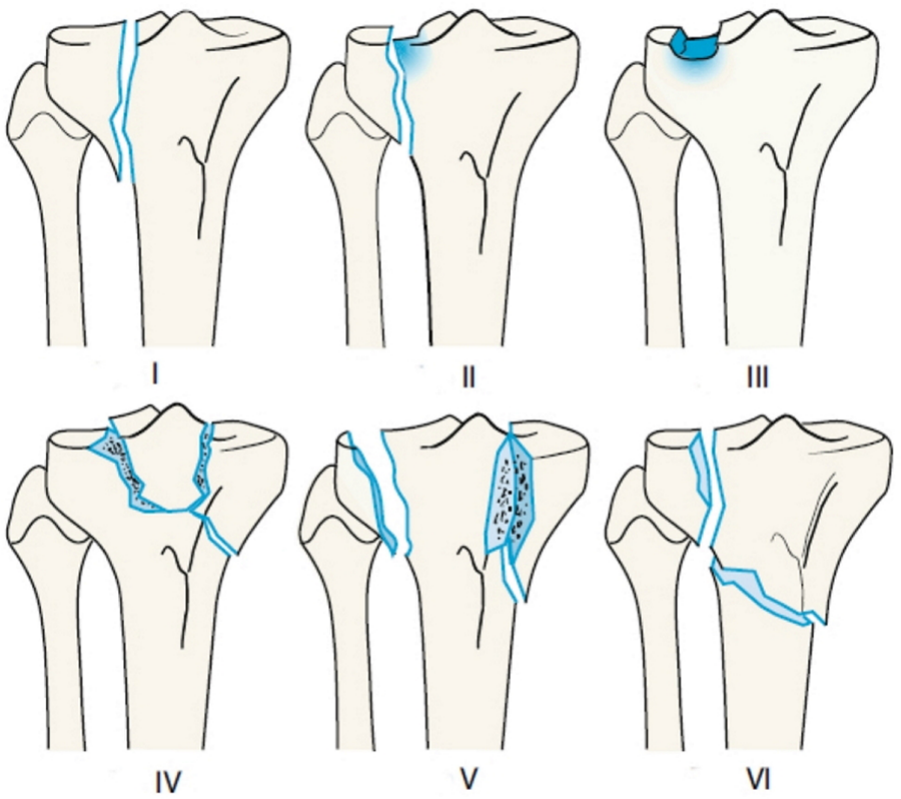
Schatzker's classification of proximal tibia fractures. (I) Wedge-shaped pure cleavage fracture of the lateral tibial plateau. (II) Splitting and depression of the lateral tibial plateau. (III) Pure depression of the lateral tibial plateau; Schatzker IIIa: with lateral depression; Schatzker IIIb: with central depression. (IV) Medial tibial plateau fracture with a split or depressed component. (V) Wedge fracture of both lateral and medial tibial plateau. (VI) Transverse tibial metadiaphyseal fracture, along with any type of tibial plateau fracture (metaphyseal-diaphyseal discontinuity). Reproduced with permission from Springer Science+Business Media. Müller-Mai CM, Ekkernkamp A. Frakturen. *Klassifikation und Behandlungsoptionen*. Berlin Heidelberg New York: Springer-Verlag (2010). 453 p.

**Figure 2:**
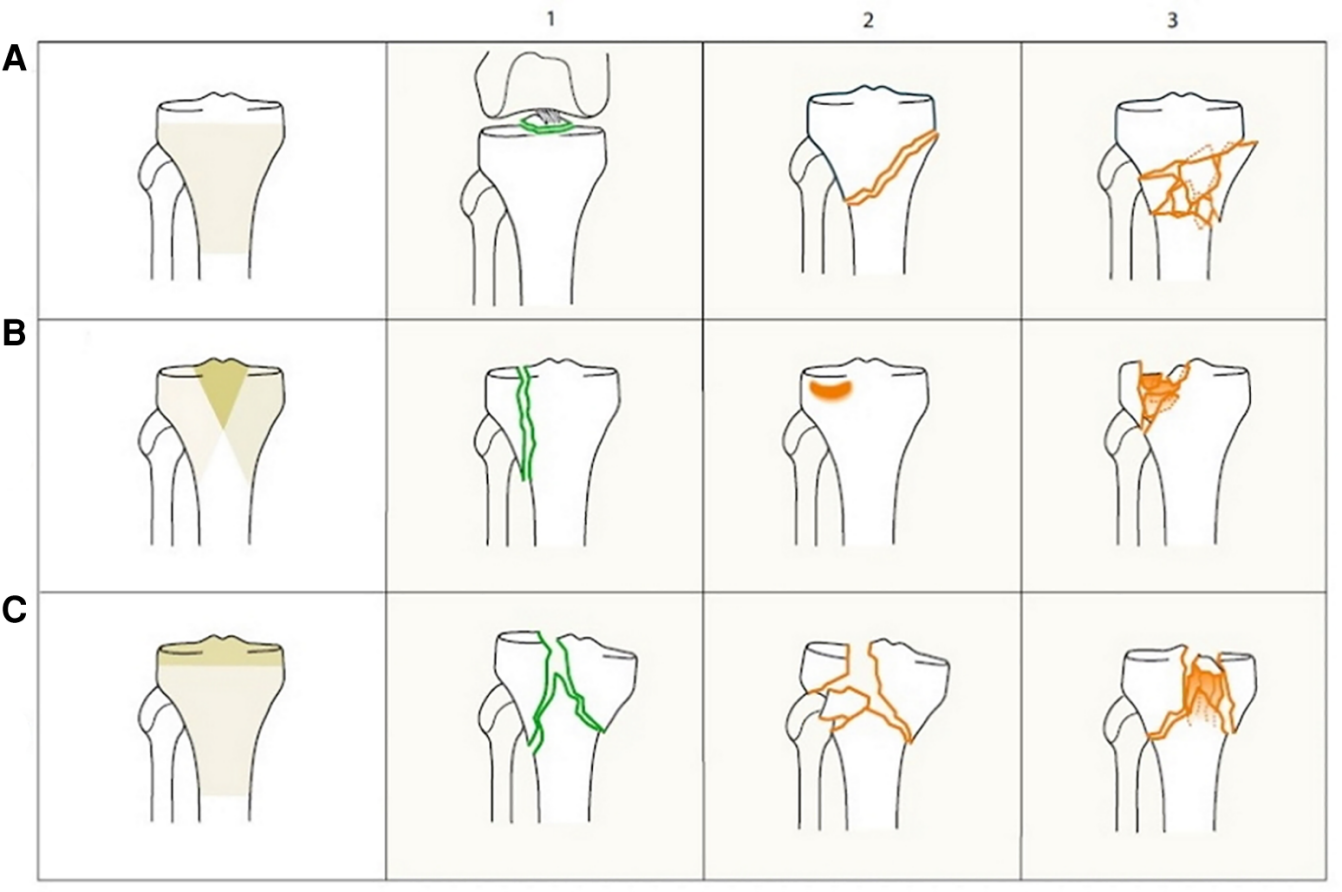
AO/OTA classification of proximal tibia fractures. 41-A extra-articular fracture. 41-A1 avulsion. 41-A2 extraarticular simple. 41-A3 extraarticular, wedge or multifragmentary. 41-B partial articular fracture. 41-B1 pure split. 41-B2 pure depression. 41-B3 split-depression. 41-C complete articular fracture. 41-C1 articular simple, metaphyseal simple. 41-C2 articular simple, metaphyseal wedge or multifragmentary. 41-C3 articular multifragmentary. Reproduced with permission from Springer Science+Business Media. Müller-Mai CM, Ekkernkamp A. Frakturen. Klassifikation und Behandlungsoptionen. Berlin Heidelberg New York: Springer-Verlag (2010). 453 p.

The classification of proximal tibial fractures has changed over the years. Schatzker et al. (1979) proposed a morphological system based on anteroposterior radiographs distinguishing six types of fractures ranging from simple split fractures to complex fractures (Figure 1) (6). This descriptive classification has gained worldwide acceptance and has been adapted by the AO/OTA (Arbeitsgemeinschaft für Osteosynthesefragen/Orthopedic Trauma Association) for their comprehensive classification in 1990 (7) (Figure 2). The AO/OTA classification distinguishes three main fractures types, A, B, and C. Group A includes extraarticular fractures as well as avulsion fractures of the intercondylar eminence. Isolated avulsion fractures of the eminence are subdivided according to Meyers and McKeever (8). Group B includes split- and depression-fractures of only one tibial condyle. Group C includes bicondylar and comminuted fractures of the tibial head.

**Figure 3:**
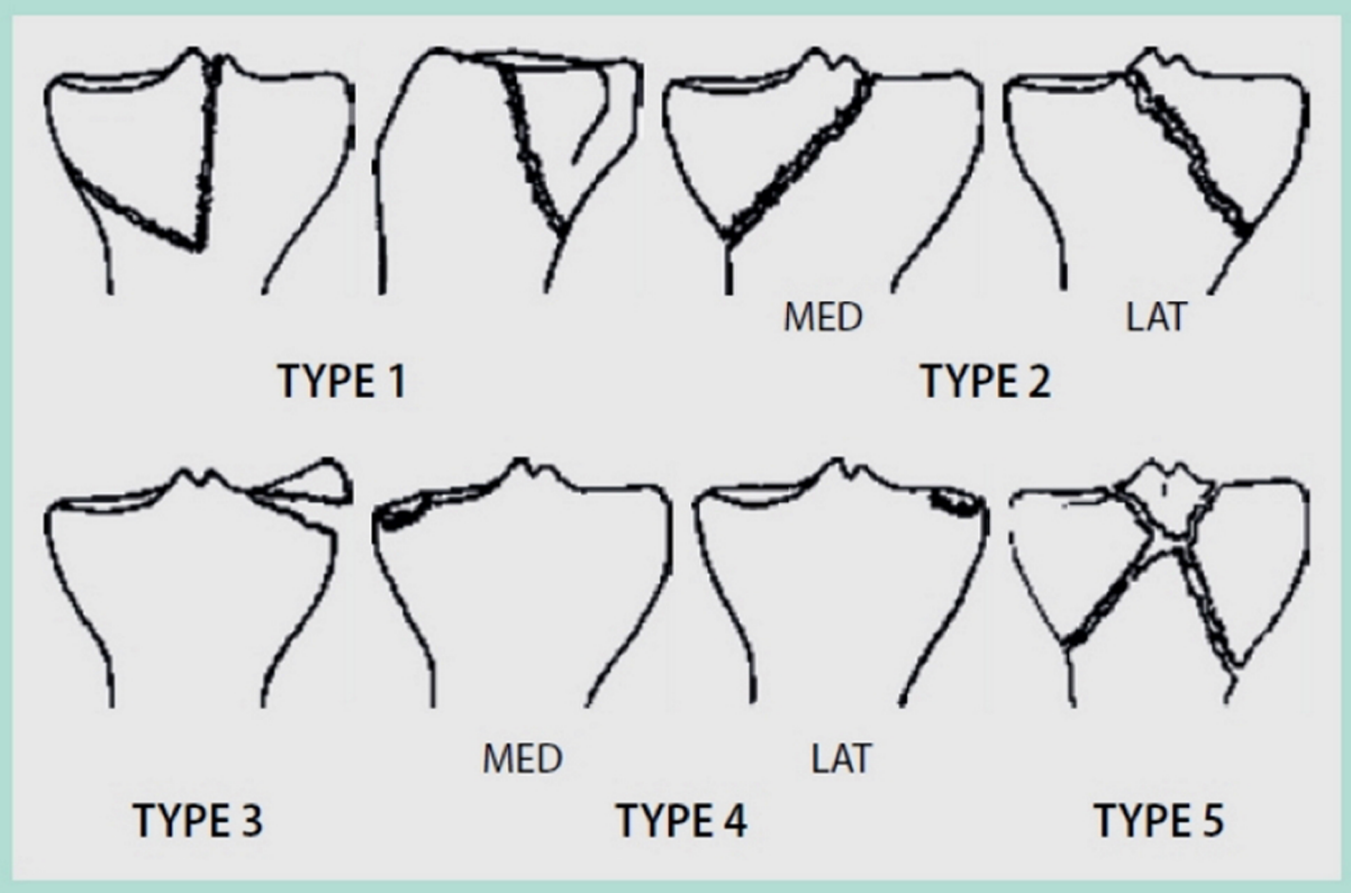
Moore's classification of proximal tibia fractures. (I) “Posteromedial split”. (II) “Entire condyle”. (III) “Rim avulsion”. (IV) “Rim impression”. (V) “Four part fracture”. Reproduced with permission from Springer Science+Business Media. Raschke M, Zantop T, Petersen W. Tibiakopffraktur [Fracture of the tibial head]. *Chirurg*. (2007) 78(12):1157–69; quiz 1170–1. German. doi: 10.1007/s00104-007-1428-z.

As early as 1981, Moore recognized the need to pay special attention to the fracture mechanism and concomitant injuries such as neurovascular and ligamentous injuries especially in fracture dislocations (9). This approach included a more three-dimensional understanding of fracture patterns (Figure 3).

• Type I describes a dorsal split fracture of the medial tibial condyle, often associated with a simultaneous rupture of the anterior cruciate ligament (ACL).
• Type II is a fracture that separates the medial (IIa) or the lateral (IIb) tibial condyle and additionally separates the intercondylar eminence by a second fracture line from the tibial shaft.
• Type III summarizes capsular avulsion fractures (lateral tibial rim fracture, Segond fragment, or avulsion of the eminence). This fracture type is often associated with ACL ruptures.
• Type IV describes the depression of the bony tibial edge including rupture of a ligament.
• Type V describes a comminuted fracture including avulsion fracture of the tibial eminence. In this case, the continuity of the attached cruciate ligaments may often be intact (“redeeming fracture of eminence”).

Tscherne et al. (10) combined elements of the classifications of Schatzker and Moore, when they introduced a new classification in 1984. Their system differentiates between plateau fractures, fracture dislocations and comminuted fractures. Tibial plateau fractures (P) are often caused by axial trauma (11) and affect the lateral plateau more frequently than the medial due to lower bone density laterally. Among plateau fractures, the classification differs between split (P1), depression (P2), split-depression (P3) and bicondylar fractures (P4). They are often seen in osteoporotic bone and are prone to occur more frequently in future due to ageing populations. Fracture dislocations (L) are caused by rotational movement and shearing. These fractures are often associated with ligamentous lesions of the femoral-tibial complex. Comminuted fractures (C) mostly originate from a high-energy trauma impact (12–14) and can result in severe damage to the tibial plateau including soft tissue damage and loss of bone.

**Figure 4:**
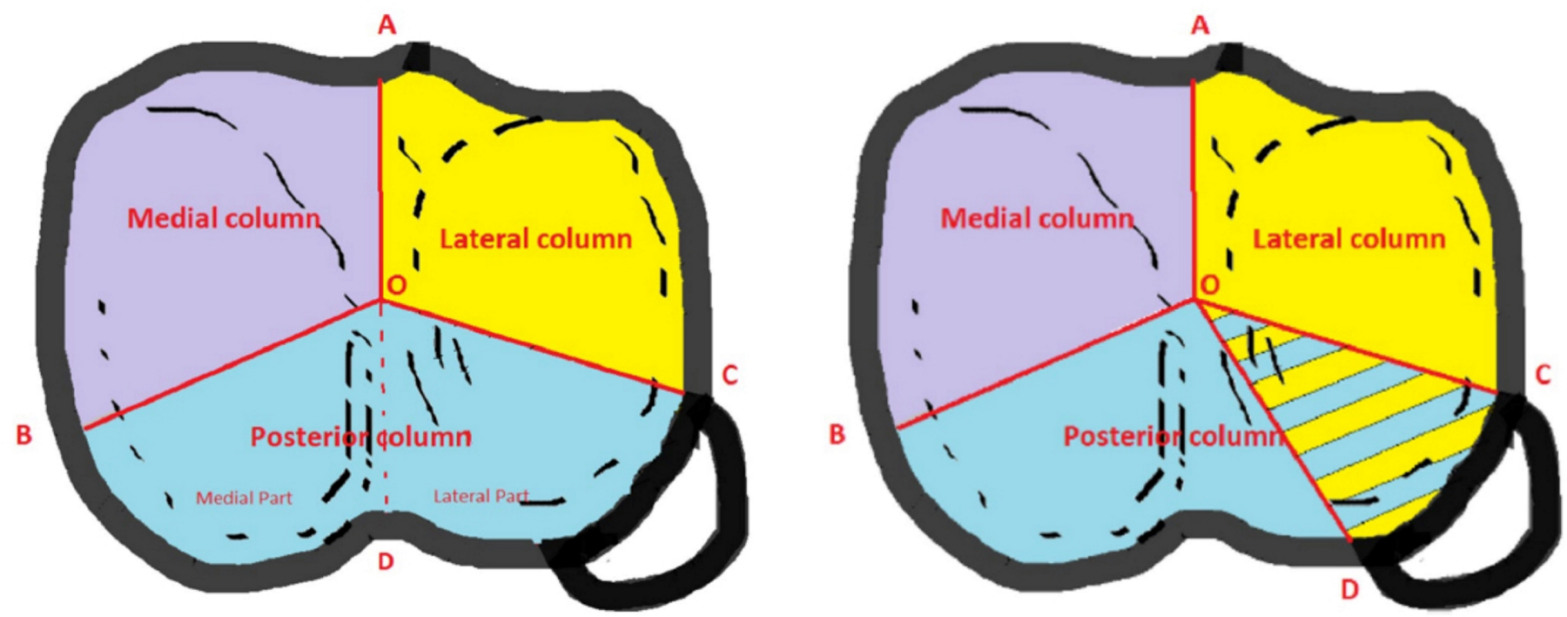
3-column concept of Luo and Hoekstra's modification. Reproduced with permission from Springer Science + Business Media. Hoekstra H, Kempenaers K, Nijs S. A revised 3-column classification approach for the surgical planning of extended lateral tibial plateau fractures. *Eur J Trauma Emerg Surg*. (2017) 43(5):637–643. doi: 10.1007/s00068-016-0696-z.

**Figure 5:**
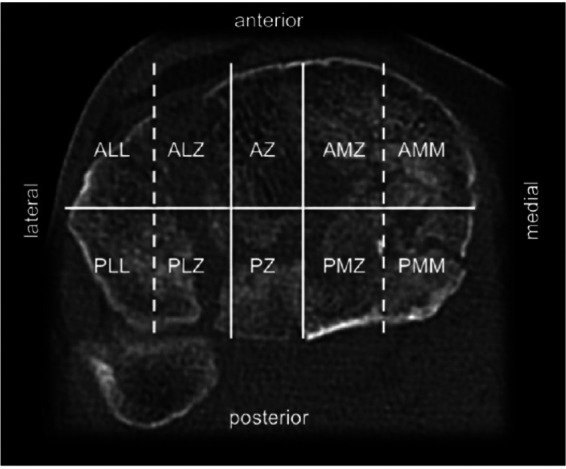
10-segment classification according to Krause Segments: ALL, anterolaterolateral; ALZ, anterolaterocentral; AZ, anterocentral, AMZ, anteromediocentral; AMM, anteromediomedial; PLL, posterolaterolateral; PLZ, posterolaterocentral; PZ, posterocentral; PMZ, posteromediocentral; PMM, posteromediomedial. Reproduced with permission from Springer Science+Business Media. Kuner E, Beeres FJP, Cagienard F, Babst R, Link BC. Reposition und Stabilisation von Tibiaplateaufrakturen: Tipps und Taktik auf Grundlage des 3-Pfeiler-Konzepts [Reduction and fixation of tibia plateau fractures: Tips and tactics based on the 3-column concept]. *Oper Orthop Traumatol*. (2020) 32(2):139–157. doi: 10.1007/s00064-020-00655-x.

Similar to other fracture regions such as spine and calcaneus, computed tomography has led to new classifications based on three-dimensional reconstructions. Two recent classifications stand out: the three-column concept of Luo and co-workers (15), later modified by Hoekstra et al. (16) (Figure 4) and the 10-segment concept of Krause et al. (17) (Figure 5). These models share the principle of three-dimensional fracture analysis mainly based on axial CT cuts below the physiological tibial plateau joint line. The difference lies in the philosophy of fracture fixation—whereas Luo et al. put the main focus on restoring the stability of the affected columns, Krause's classification places the reconstruction of the damaged joint surface at the center of attention.

Luo et al. introduced a concept of affected columns similar to thoracolumbar or acetabular fractures. Depending on the presence of at least one separate fragment in the respective column they proposed a technique of fixation of all three columns in complex fractures (15). This concept has later been extended to the fixation of zero-, one- or two-column fractures (18). Hoekstra emphasized the special role of the so-called posterolateral corner into which lateral column fractures can extend. This section is bounded by the center of the tibial plateau and the anterior and posterior borders of the fibular head. According to Luo's concept, it belongs to the posterior column, but—using a variable angle locking compression plate—the posterolateral corner (PLC) may also be addressed *via* an (antero-) lateral approach (16).

In contrast, the 10-segment concept of Krause et al. aims at best access to the affected articular surface. Starting with four quadrants, they divided the tibial plateau into ten segments consisting of two segments per quadrant plus the anterocentral and posterocentral segment for avulsion fractures of the eminentia (17). The authors could show that posterior segments were most frequently affected in OTA/AO type B and C fractures. However, visualization of the posterior articular surface is limited, even through extended surgical approaches (19). Visualization may be improved by “fracturoscopy”, which is particularly recommended for fractures of the posterolaterocentral segment (20).

The ideal classification of proximal tibial fractures has not yet been found. The OTA/AO and Schatzker's classifications have worldwide recognition, but they do not consider accident mechanisms and concomitant injuries. Furthermore, being based on anteroposterior roentgenograms, they do not use the additional information provided by computed tomography or magnetic resonance imaging and hence are underrating certain fracture types. More recent classifications based on computed tomography try to improve three-dimensional understanding. In addition to an improved detection of posterior fracture types these classifications facilitate the choice of approach (19).

### 2.2. Diagnostics

An essential step in diagnostics must be the inspection and evaluation of open or closed soft tissue damage, classified according to Tscherne and Oestern (14), which is the key procedure for planning further treatment strategies.

For open fractures, the classification according to Gustilo and Anderson is also widely used (21). Special attention must be paid to evolving compartment syndrome and, in terms of dislocation, potential harm of vascular and neurovascular structures among the popliteal region, which requires detailed assessment and documentation of neurovascular status. In case the proximal fibula is involved in the tibial fracture, the peroneal nerve might also be affected. Perfusion status and ultrasound documentation should be performed, especially in fracture dislocations. Fractures of the proximal tibia may cause compartment syndrome, especially when the fracture extends into the tibial shaft. Measurement of the compartment pressure can be performed; however, a compartment syndrome is basically a clinical diagnosis which requires immediate decompression of all compartments of the lower leg.

Conventional radiology (x-ray) of the knee joint and proximal lower leg in anterior-posterior and lateral planes, usually allows immediate evaluation of the underlying fracture type. Nevertheless, computed tomography provides the most detailed illustration of the fracture in all dimensions and therefore should be used in more complex fractures. In addition, two (2D) and three-dimensional (3D) reconstructions improve reliability of classification and facilitate the planning of any therapeutic procedure (22).

In knee dislocations with suspected arterial lesions or in case of an ankle-brachial-index (ABI) of <0.9, (CT-) angiography is mandatory, because even patients with an intact pulse status show intimal lesions in about 9% of cases, as Howells et al. could show (23).

In conventional x-rays, recognizing barely dislocated edge fragments in ligament trauma may be difficult. In this situation MRI (magnetic resonance imaging) is helpful. MRI enables recognition of the impact zone (“bone bruise”, representing edema underneath the cartilage layer) with undislocated fragments. It also allows assessment of the integrity of intra- and extraarticular soft tissue structures such as: menisci, cruciate and collateral ligaments. The MRI is a widening of the diagnostic spectrum in cases of doubt, as it is mostly impossible to evaluate the stability of the knee joint from a clinical point of view during the initial phase. It was furthermore shown to increase the interobserver agreement on fracture classification and operative management of tibial plateau fractures (24).

Nevertheless, even plain radiographs alone can make a valuable contribution to diagnosis of soft tissue injuries. Widening and depression of the lateral plateau, as measured on plain radiographs, correlate with the incidence of soft tissue injury as detected on MRI (25). Gardner et al. found lateral meniscus injuries in 83% of fractures, when the point of maximum joint depression compared to the plane of the preinjury joint line was greater than 6 mm or widening displacement (using the lateral femoral condyle as a reference) was greater than 5 mm. The reported incidence of capsuloligamentous and meniscal injury approached 30% with increasing displacement of fragments (25). Using multislice detector computed tomography (MDCT) scans for measuring depression and widening, the probability of soft tissue injuries can be estimated even more precisely. With every 1.0 mm of lateral plateau widening the risk of lateral meniscus and fibular collateral ligament injuries rises to 40% and 32%, respectively (26).

In summary, besides conventional x-ray, CT scanning is the standard for evaluating bony injury including articular depression. It depicts osseous avulsions with a high sensitivity and specificity and can exclude ligament injuries with a high negative predictive value. MRI is the standard for evaluating associated soft tissue injury, such as meniscal, ligamentous, or chondral injury, in association with fractures of the tibial plateau (4, 27). Because of the high incidence of concomitant meniscal and ligamentous injuries in patients with lateral tibial plateau fractures, Kolb and co-workers recommend MRI in those patients (26).

### 2.3. Therapy

There are three aims in the therapy of proximal tibial fractures:

1. Reconstruction of the joint surface: The tibial joint surface should be restored as accurately as possible; should uneven levels persist, there is a risk of elevated impact force and accelerated erosion leading to secondary osteoarthrosis, particularly in the region of maximum weight bearing without meniscal cover.
2. Reconstruction of knee axis and a “height stable’ tibial plateau: Perfect articular congruency is sometimes difficult to achieve, especially in highly comminuted fractures. Furthermore, there is no consensus on the tolerable step-off in articular surface. Compared to other joints, articular incongruities alone seem to be relatively well tolerated in the tibial plateau. Besides joint stability and coronal alignment, special attention should be paid to retention of the meniscus (28). Joint instability or “pseudolaxity” due to reduced height of the tibial head rather than ligamentous injury, is strongly associated with poor outcome (29).

Another mechanism for posttraumatic accelerated degeneration may be the mechanical overstressing of one condyle due to an incorrect mechanical axis of the lower limb (varus—valgus deformity with lateral shift of the mechanical axis). Deviation of the anteroposterior axis results in restricted extension due to elevated posterior slope or leads to genu recurvatum.

3. Early mobilization: Prolonged immobilization of the joint means deterioration of the cartilage nutrition which is already impaired. Arthrofibrosis is another result of prolonged immobilization.

Any therapeutic strategy depends on the type of fracture and the collateral soft tissue damage. In terms of operative planning and access, stable soft tissue surroundings must be achieved first. For this purpose, intense swelling and contusion should be treated with immobilization and decongesting measures such as elevation and lymph drain pumps. Attention must be paid to the possibility of compartment syndrome which would then require early diagnosis and therapy. The decision for decompression of all tibial compartments should be taken swiftly.

High-grade unstable fractures may require a temporary external fixation for providing regular anatomical geometry and decompression of the proximal tibia. This procedure is recommended particularly after high-energy trauma impact and multiple trauma (13). Following the practice of “span-scan-plan” the first step is external fixation with monolateral carbonfiber fixation systems (“span”) which allow radiographic access to the fracture and improve interpretation of radiological imaging while avoiding setting pins in the area of the later surgical approach. After imaging with MDCT and/or MRI (“scan”) the definitive surgical strategy (timing, approach) is determined mainly by concomitant soft tissue injuries and fracture characteristics (“plan”) (30). Open fractures demand debridement and lavage. Nevertheless, a second look procedure is typically necessary after 48 h. In cases of injury affecting extension of the knee (i.e., fracture of tibial tuberosity, rupture of patellar tendon), the reconstruction of these structures is indicated during the first surgical approach. Insufficient restoration of extension apparatus may cause a secondary loss of reduction, even when external fixation was applied.

#### 2.3.1. Conservative treatment

Conservative therapeutic approaches are viable for single, non-dislocated fractures. As mentioned above, there is no consensus about the acceptable degree of articular step-off and articular gap. Usually incongruencies of less than 2 mm or gaps under 5 mm are considered tolerable (31).

**Figure 6:**
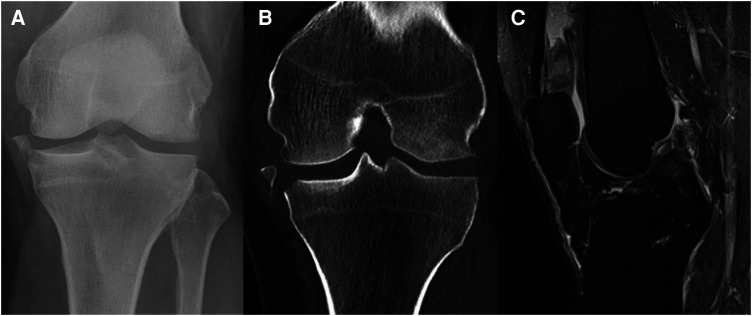
Case 1, 54y male patient, motorcycle accident. AP radiograph (**A**) and coronal CT reconstruction (**B**) showing a Moore type III medial avulsion fracture, MRI radiograph (**C**) showing the concomitant ACL rupture.

None or barely dislocated tibial edge fragments (posteromedial or anterolateral edge fragment, Segond fracture) are indications for conservative therapy. Nevertheless, these fractures are often accompanied by ligament lesions which may be underdiagnosed and must be evaluated. The initial diagnostic work-up is characterized by only limited clinical examination of knee and tibia (pain, risk of dislocation) and requires an extended diagnostic effort such as MRI imaging. Collateral lesions such as cruciate or collateral ligament defects should be reconstructed primarily or secondarily, depending on the respective indication and collateral injuries (see Case 1, Figure 6).

#### 2.3.2. Arthroscopic reduction and internal fixation (ARIF)

**Figure 7:**
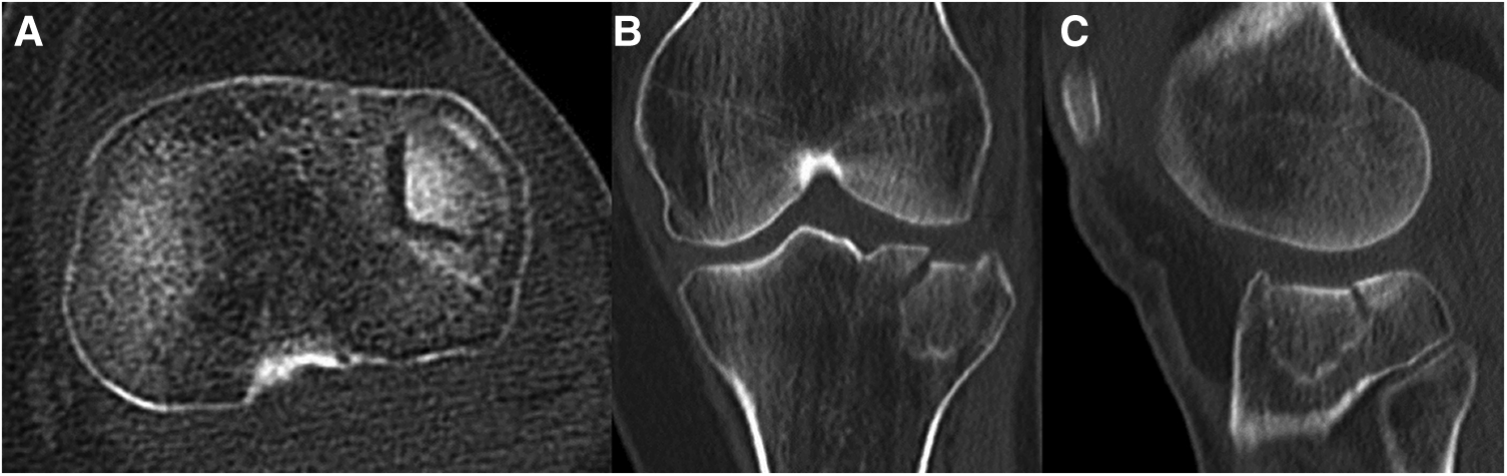
Case 2, 65year female patient, low energy knee distorsion in domestic environment. Lateral tibial plateau impression fracture (Schatzker IIIa, AO 41-B2, Zero-column, anterolaterocentral segment), as seen on the axial CT cut (**A**), coronal (**B**) and sagittal (**C**) reconstruction.

Arthroscopically assisted surgical procedures are indicated for non to barely dislocated split fractures, depression in the mid or posterior joint region (e.g., AO A1, B1 to 3, Schatzker I, II, III; see also Case 2, Figure 7) and for avulsion fractures of the intercondylar eminence (4, 32).

**Figure 8:**
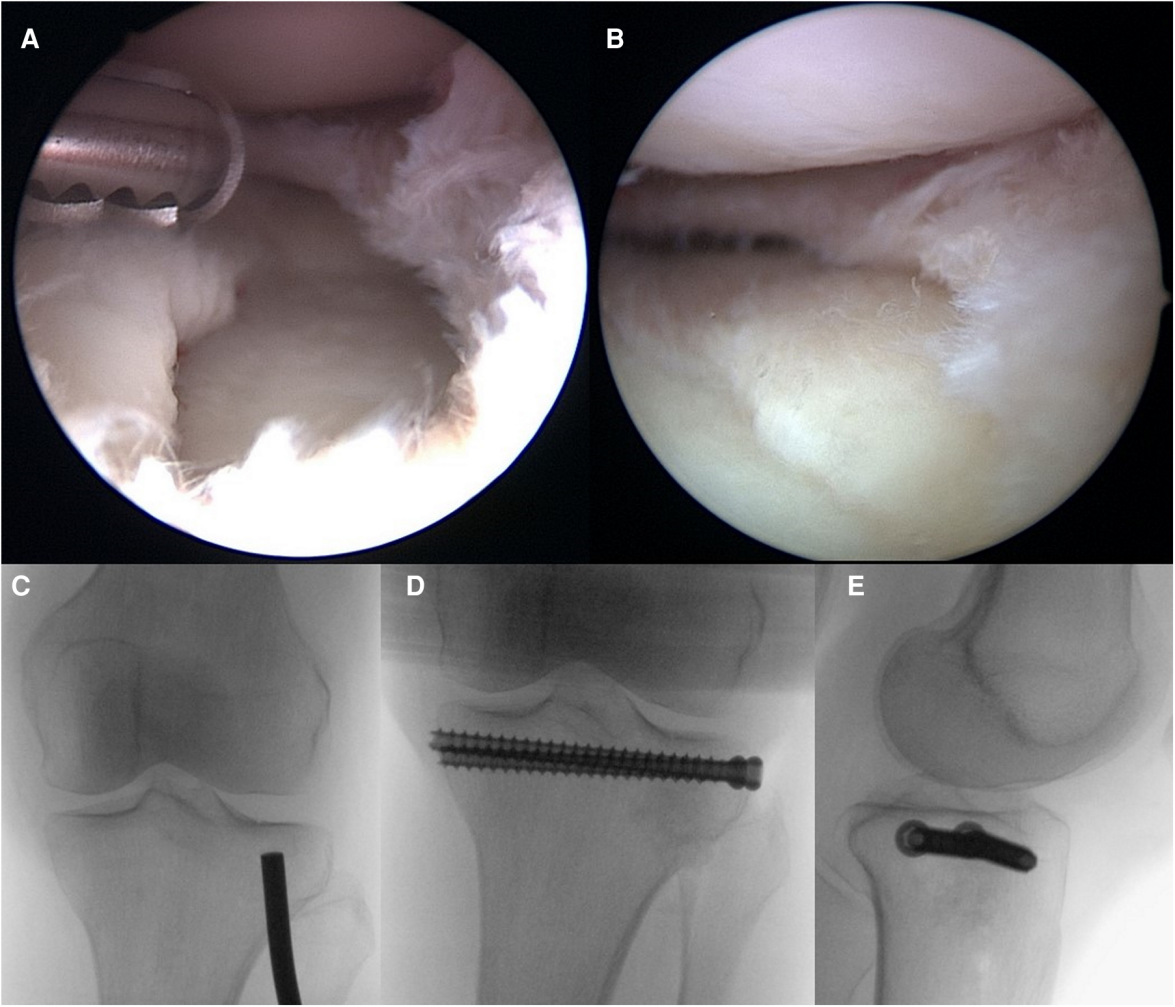
Case 2, arthroscopic view before (**A**) and after (**B**) reduction, flouroscopic view of reduction with a bone tamp (**C**) followed by allograft as bone substitute and interal fixation, as seen in the AP (**D**) and lateral (**E**) view.

Arthroscopically assisted osteosynthesis is, in general, thought to be favorably compared to open procedures (4, 33–35) for certain reasons:

1. Small joint incisions for arthroscopy allow reduction and internal fixation with minor collateral damage to surrounding soft tissues and thus fewer soft tissue complications (36). Lower morbidity allows earlier postoperative rehabilitation and mobilization. Hospitalization and incidence of postoperative arthrofibrosis can be decreased compared to open procedures.
2. Compared to the fact that 2D fluoroscopy can only detect a step-off of 5 mm and more (37), arthroscopic visualization allows precise evaluation and exact staging of joint surfaces see Case 2, Figure 8.
3. Furthermore, concomitant extra- and intraarticular defects (i.e., cruciate ligaments, menisci) may be treated in the same session (e.g., refixation of meniscus).

The existing literature is controversial regarding these advantages. The studies including Schatzker I–III fractures found equal or superior results of ARIF compared to ORIF regarding the above-mentioned advantages e.g., complications (38), shorter hospital stay (39), lower infection rate (40), better PROM (KSS score) (41), and radiological outcome (Rasmussen's radiological score (38). ARIF was also superior compared to ORIF in a study including Schatzker I–IV fractures regarding radiological outcome (Rasmussen's radiological score) (42).

A further advantage of arthroscopy lies in the treatment of intraarticular soft tissue injuries. A variety of soft tissue injuries, e.g., meniscus and ligament tears, are common in patients with tibial plateau fracture (>70%) and can be diagnosed as well as treated utilizing arthroscopy. Menisci are the most injured soft tissue structure in 57%–80% of patients in both Abdel-Hamid's (43) operatively and Shepherd's non-operative patient collective (44). These traumatic tears occur mainly in the meniscal periphery, a region very well suitable for repair, which helps for the long-term outcome regarding OA and return to previous physical activities (37). Tears of the anterior cruciate ligament were reported to range between 10% to 25% by Bennett (36) and Abdel-Hamid (43). Tears of the posterior cruciate ligament, tibial or fibular collateral ligament, and fibular nerve were reported to range in between 1% to 5% of patients with tibial plateau fractures. Injuries of the popliteal artery occurred even less frequently in both studies (36, 43).

Especially meniscus and ACL tears have a high incidence in Schatzker type IV fractures (split-depression fracture of the medial plateau), whereas MCL injuries are more common in type II fractures. Nevertheless, type II fractures had the highest prevalence of concomitant soft tissue injuries (36, 43). This confirms that soft tissue injuries are not just found among non-dislocated or minimally displaced tibial plateau fractures (44). If untreated, these may account for later development of secondary osteoarthritis especially in the young and active patient. While MRI provides the benefit of detecting these lesions non-invasively, arthroscopy gives a detailed “real-time” impression of the intraarticular situation and the chance of repairing or reconstructing the injured soft tissue structures, if suitable. And even if MRI has a high accuracy, there are still pathologies missed in the MRI and its report. Concomitant lesions such as ligament or meniscal tears can be subjected to repair during one session, especially in case of non-dislocated fractures treated conservatively. ACL or PCL tears are in general reevaluated after the bone stock has healed; and if necessary, then treated.

Nevertheless, arthroscopic technique has also its limitations e.g., it is not suitable for complex fractures. The draining of irrigation fluid *via* fracture split or capsule defect may cause severe swelling of the surrounding tissue up to iatrogenic compartment syndrome (45), even though this a very rare complication. When using arthroscopy in the treatment of tibial plateau fractures, it is recommended to abandon automatic arthroscopic pumps. The so called “fracturoscopy” method, which has been described by Krause et al., consists of direct arthroscopic visualization and “live” fracture reduction of complex tibial plateau fractures without any fluid or with less than 30 mmHg of fluid pressure. Risk of compartment syndrome is low while detection of malreposition is superior to fluoroscopy especially in the posterolaterocentral segment according to the 10-segment-classification (20).

#### 2.3.3. Closed reduction and percutaneous fixation (CRIF)

Osteosynthesis of simple fractures (i.e., split/depression fractures AO 41 B1 to B3) without larger bone defect can be achieved by percutaneous cannulated screw fixation (see Case 2, Figure 8). In accordance with the previous chapter, simultaneous arthroscopic control of joint surfaces would be desirable if tolerated by surrounding soft tissue conditions.

With balloon tibioplasty there is another minimally invasive technique showing promising early results. As in spine surgery fracture, reduction is achieved by inflating a balloon with a radiopaque dye under fluoroscopic (and/or arthroscopic) control. Compared to a conventional bone tamp, the balloon does not implicate fenestration and had less risk of joint penetration in cadaveric model (46, 47). Nevertheless, as with many new techniques there is a steep learning curve. Balloon failures, extravasation of bone fillers or failures to elevate the depressed articular fragment were observed and require a backup plan (48). Randomised controlled trials comparing tibioplasty with traditional methods of fracture reduction are in progress (49, 50).

#### 2.3.4. Definitive treatment by external fixation

While monolateral bridging external fixation is common as a temporary tool in a staged management of unstable fracture types, external fixation with an Ilizarov ring fixator or hybrid fixator is suitable for definitive treatment, either with or without additional osteosynthesis (51–53). In highly unstable fractures (e.g., AO 41 C1 to 3, Moore V, Schatzker V and VI), two additional rings in the distal femur with hinged rods to bridge the knee are needed. This technique combines the advantages of soft tissue protection with high stability allowing full weight bearing (54).

In terms of complications, pin track infections are most frequently observed. They are superficial infections and do not require a change of the corresponding pin. However, proximal pins should be inserted with regard to the joint capsule anatomy (subchondral cartilage layer) to avoid pin track-associated joint infections. Patient's compliance is an important precondition. The most proximal pin should have a minimum safety distance of 20 mm from the joint line, as the synovial layer measures joint capsule inserts up to 14 mm below tibial plateau (55). Another pitfall in placing fine wires is damage to neurovascular structures, particularly the peroneal nerve (56).

In summary, patients with a high-energy fracture of the tibial plateau treated with external fixation have a good prognosis for satisfactory knee function and low rate of severe osteoarthritis in long-term perspectives (51, 57). Typical indications for bridging of the knee joint are severe soft-tissue damage, ligamentous damage, poor bone quality and arterial injury (52).

#### 2.3.5. Open reduction and internal fixation (ORIF)

The two factors determining knee function and risk of osteoarthritis in the long-term are: (1) ligamentous and osseous stability with correct mechanical axis, and (2) reconstruction of joint surfaces. ORIF should be applied when the above mentioned less invasive alternatives are not suitable to reach these main treatment goals.

**Figure 9:**
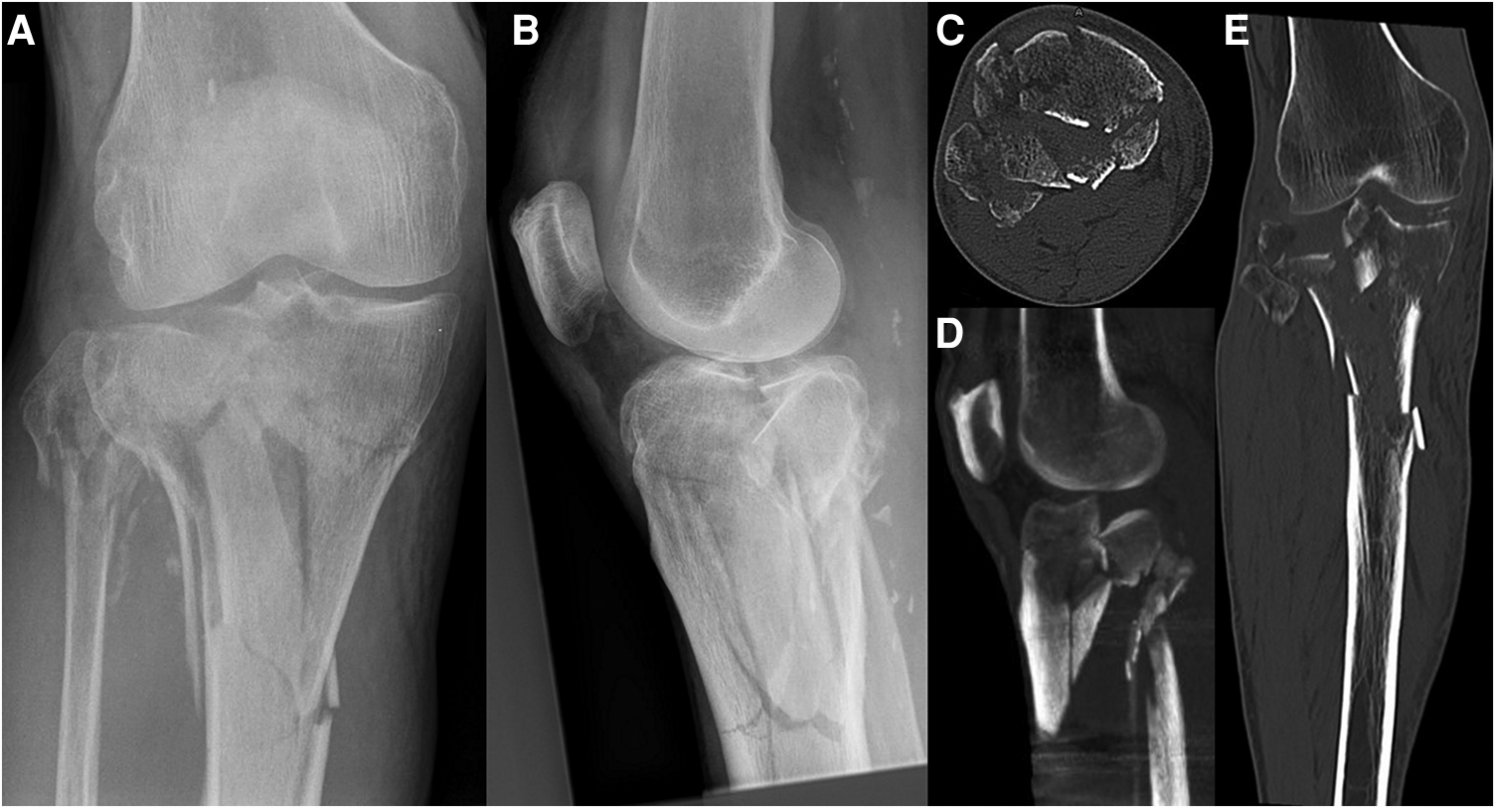
Case 3, 64 year male patient, homeless person, alcohol-related fall onto track bed. Comminuted 3-column fracture extending into diaphysis with initial valgus deformity, closed soft tissue damage grade II according to Tscherne and Oestern, uncooperative patient. AP (**A**) and lateral (**B**) view, axial CT cut (**C**), coronal (**D**) and sagittal (**E**) reconstruction.

**Figure 10:**
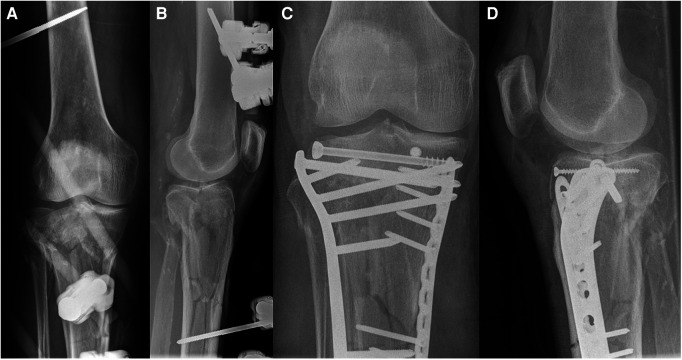
Case 3, comminuted 3-column fracture, AP (**A**) and lateral (**B**) x-ray after bridging external fixator (arterial injury ruled out by CT angiography), AP (**C**) and lateral (**D**) view after CRIF of articular fracture and MIPO of extraarticular fracture.

However, as in other fields of orthopedic trauma surgery, soft tissue management has top priority. Even in cases with minor trauma impact careless handling of surrounding tissues can cause severe complications concerning wound healing, infections and delayed bony union. Accordingly, recent developments in the treatment of tibial plateau fractures imply a differentiated practice using a large variety of special approaches suitable for any given injury pattern with minimal threat to surrounding soft tissues and periosteal nutrition. Especially in comminuted fractures with severe soft tissue damage, extended approaches should be avoided whenever possible. To reach both reduction of articular surface and restoration of mechanical axis, a limited open access to the joint can be combined with a minimally invasive closed reduction and internal fixation of the metaphyseal fracture component by a bridging angular stable plate see Case 3, Figures 9 and 10.

In recent years, the CT-based three column concept of Luo et al. (15) and its derivative classifications (16, 18) have proven beneficial in preoperative planning including bedding of the patient, surgical approach, implant type and use of bone allograft. Understanding the injury mechanism is of paramount importance for the correct choice of approach and implant positioning.

Accordingly, this system is used in the following to provide an overview of common strategies in open reduction and internal fixation of tibial plateau fractures.

In One, Two or Three-column fractures the position of the knee at the time of injury (flexion/extension) and the direction of the deforming force (varus/valgus) can be derived from measuring the posterior tibial slope angle (pTSA) and the medial tibial plateau angle (mTPA) (18).

##### 2.3.5.1. Zero column fracture

This group of impression fractures (Schatzker III, AO 41 B2) can be successfully treated minimally invasively under arthroscopic and/or fluoroscopic visualization (see above). When choosing ORIF most commonly a limited anterolateral approach is used for tapping of the subsided plateau fragment *via* a small cortical window.

##### 2.3.5.2. One column fracture

###### 2.3.5.2.1. Lateral column fracture

As these fractures are mainly caused by axial and valgus forces in extension, fracture fixation should buttress the lateral column against secondary valgus deformity, for which a standard anterolateral approach is suitable. In pure split fractures (Schatzker I, AO 41 B1), percutaneous screw fixation alone is an alternative option. In this case, usually two or more screws are placed just below the joint surface and one at the fracture apex.

###### 2.3.5.2.2. Medial column fracture

Accordingly, isolated medial column fractures are typically caused by axial and varus forces in extension. As the choice of approach is determined by the location of the main split-wedge fragment, a medial column fracture is best addressed by an anteromedial or medial approach.

###### 2.3.5.2.3. Posterior column fracture

In contrast to medial and lateral column fractures, the rare isolated posterior column fracture results from axial force applied to the knee in flexion. Depending on the location of the fracture apex seen on axial CT, possible approaches are posteromedial, direct posterior [e.g., Carlson, Lobenhoffer (12, 58)] or posterolateral.

##### 2.3.5.3. Two column fracture

###### 2.3.5.3.1. Medial and posterior column fracture

These fractures are caused either by varus forces in extension or flexion, the latter leading to more complex injuries involving the lateral aspect of the posterior column. Depending on the amount of posterior column involvement, either a medial approach (extension injuries) is still sufficient or a posteromedial approach (flexion injuries) is preferred. Using the extended posterior approach according to Luo et al. exposure is possible up to the fibular head.

###### 2.3.5.3.2. Lateral and posterior column fracture

**Figure 11:**
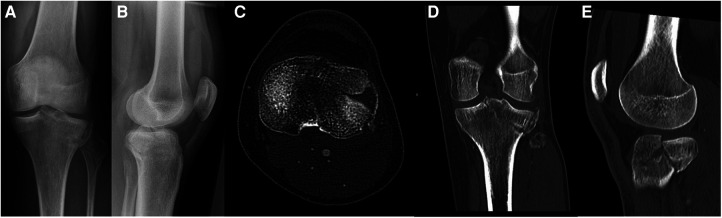
Case 4, 30 year male patient, syncopal fall. Posterolateral split impression fracture, AP (**A**) and lateral (**B**) view, axial CT cut (**C**), coronal (**D**) and sagittal (**E**) reconstruction (Schatzker IV, AO 41-B3, Moore Type II lateral, posterolateral 2-column fracture, segments involved: ALL, PLL, PLC, AC, PC).

**Figure 12:**
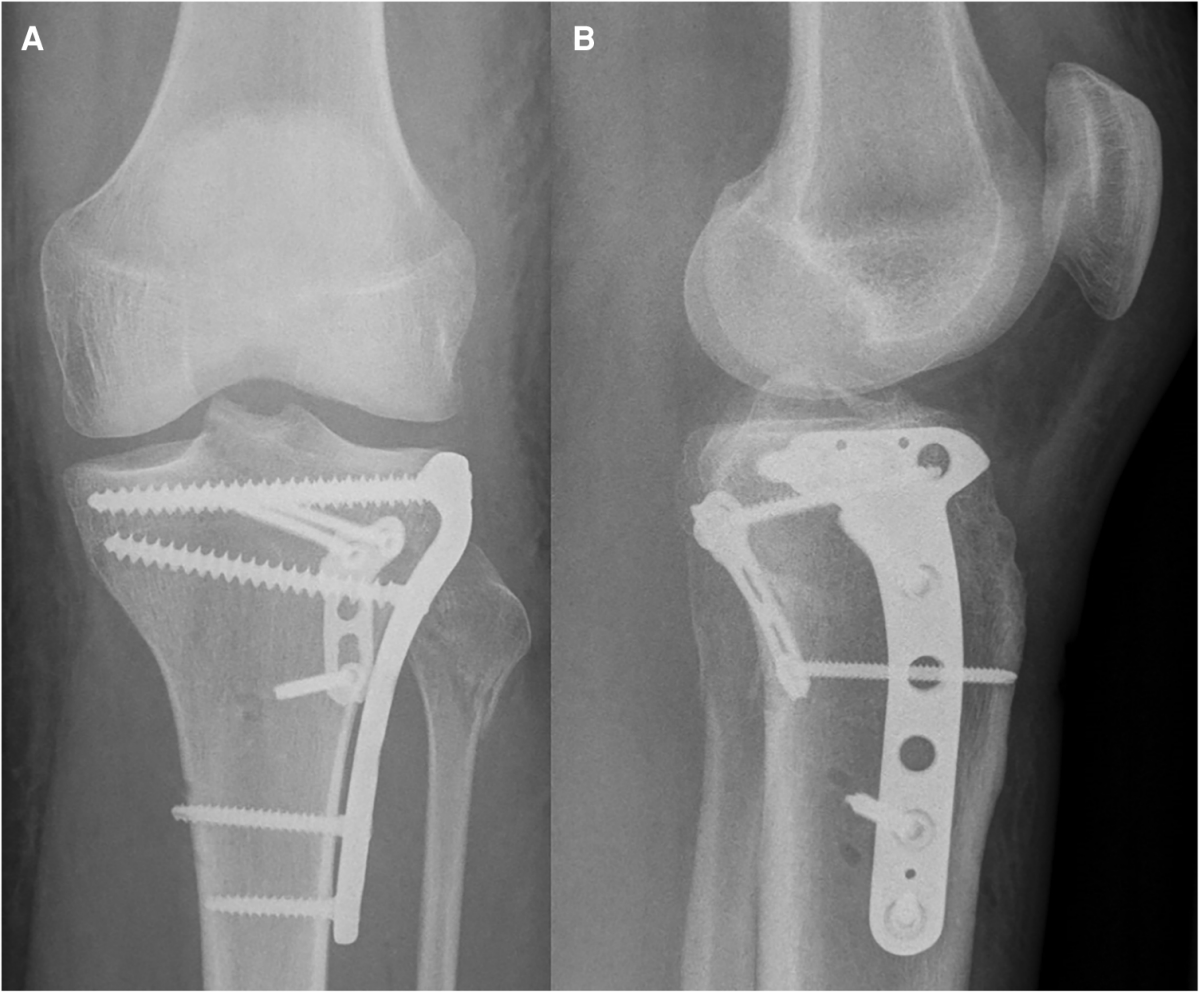
Case 4, posterolateral split impression fracture, postoperative AP (**A**) and lateral (**B**) x-ray after ORIF with two angular stable plates using the frosch approach.

This relatively common combination is caused by valgus forces. Again, the more complex injuries usually occur in knee flexion, often requiring posterolateral additional to the (antero-)lateral buttressing. Modern approaches such as the Frosch approach (59) allow sufficient exposure of the posterolateral plateau without osteotomy of the fibular head (see Case 4, Figures 11, 12). However, with this approach, exposure of the posterior column is limited to the posterolateral region. Luo and colleagues proposed the combination of an anterolateral and an extended posterior approach in the so-called floating position enabling both approaches without changing position in between.

###### 2.3.5.3.3. Medial and lateral column fracture

This fracture type is usually caused by axial force in knee extension. Classical anteromedial and anterolateral approaches are the method of choice for medial and lateral buttressing.

##### 2.3.5.4. Three column fracture

**Figure 13:**
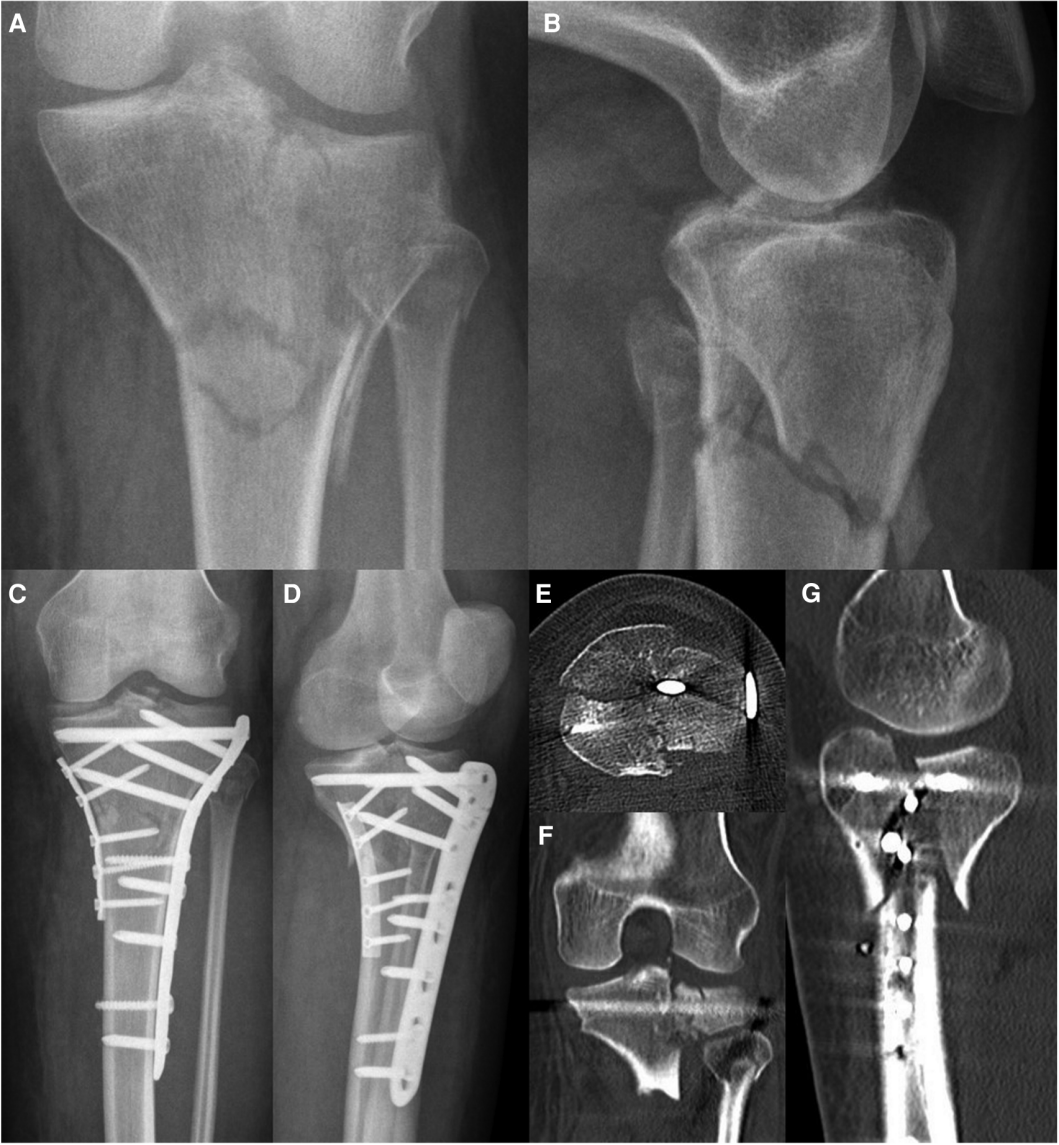
Case 5, 27 year female patient, motorcycle accident during holidays. 3-column fracture (Schatzker VI, AO 41-C3, Moore V, 3-column-fracture) displayed by AP (**A**) and lateral (**B**) x-ray. Postoperative AP (**C**) and lateral (**D**) x-ray as well as axial CT cut (**E**), coronal (**F**) and sagittal (**G**) reconstruction after first surgery abroad (ORIF by double plate fixation *via* anterolateral and medial approaches) reveal a persisting dislocation of a large posteromedial fragment causing an articular step-off of more than 5 mm.

**Figure 14:**
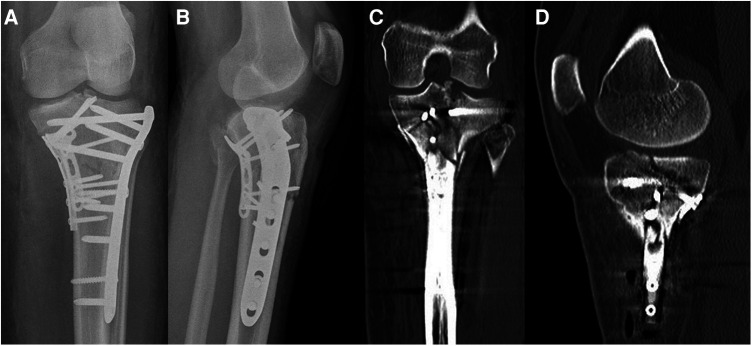
Case 5, 3-column fracture after revision surgery (posteromedial extension of the pre-existing medial approach, direct reduction and fixation of the split wedge fragment with a 3.5 mm T-plate), AP (**A**) and lateral (**B**) x-ray, coronal (**C**) and sagittal (**D**) CT reconstruction.

**Figure 15:**
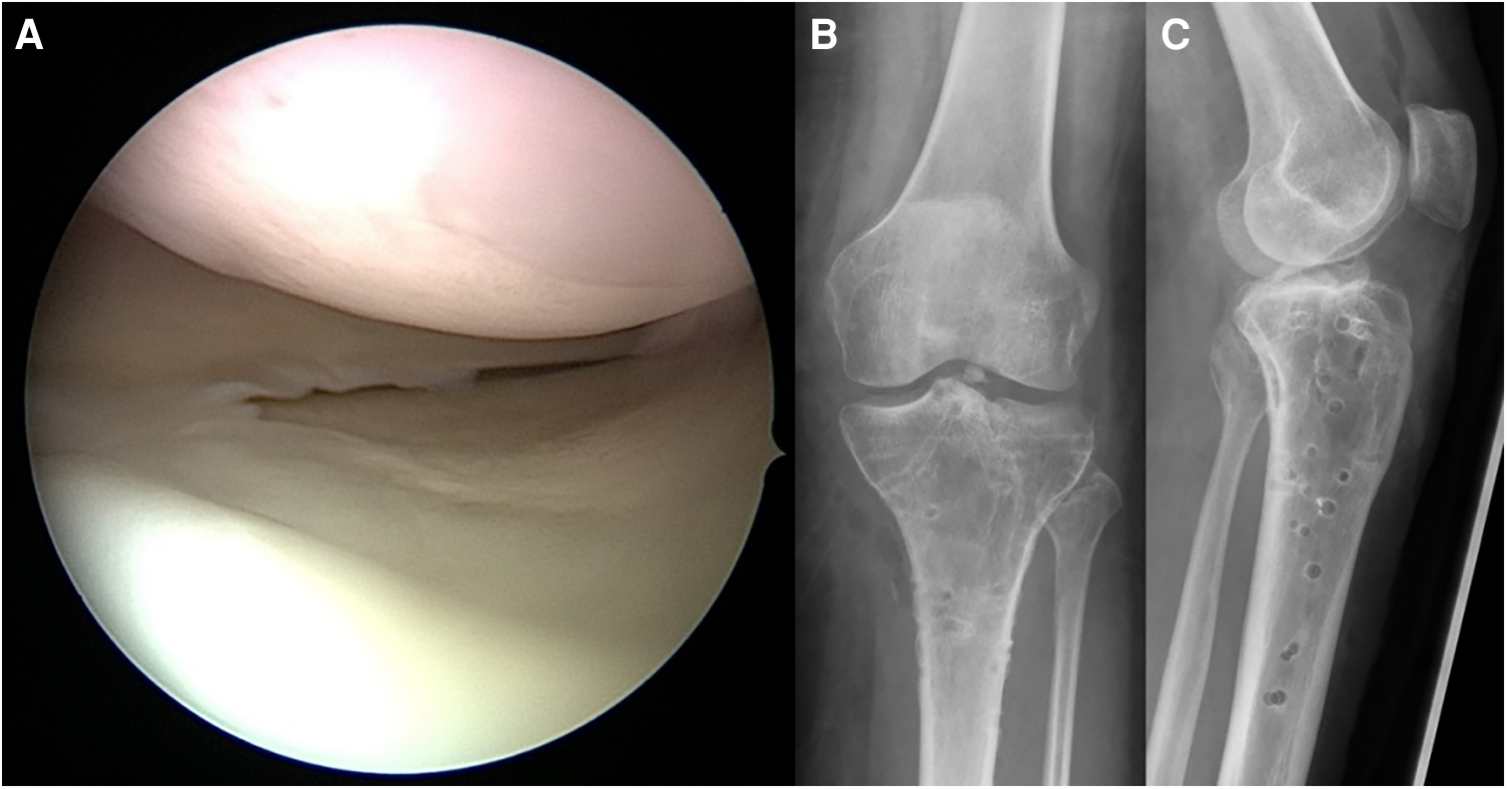
Case 5, 3-column fracture, arthroscopic view (**A**) and AP (**B**) and lateral (**C**) x-ray after implant removal 16 months later demonstrating restoration of mechanical axis and joint surface.

This most complex of fracture types can be caused by several combinations of forces acting on the knee in extension or flexion. Treatment principles are similar to the therapy of two-column injuries with each of the three columns addressed separately (see Case 5, Figures 13 to 15). In the updated Three Column Fixation concept (uTCC) according to Luo et al. the authors recommend identifying the main acting force and the position of the knee at the time of injury by measuring the pTSA (medial and lateral) and mTPA as mentioned above. For example, in an injury with a dominating varus force indicated by a negative medial Tibial Plateau Angle acting on the knee in flexion shown by a negative posterior Tibial Slope Angle, the so-called main buttressing plate should be positioned posteromedially (=compression side) as a first step. Additional smaller buttressing plates may be needed to address independent secondary column fractures. The opposite (tension) side should be stabilized with a supporting plate, usually as the last sequence in reconstruction. In some cases, the tension side will show additional ligament ruptures or bony avulsions which might also need surgical reconstruction.

While the uTCC according to Luo et al. puts the focus on the reconstruction of the mechanical axis and joint stability, the 10-segment system of Krause and co-workers focuses on the reconstruction of the joint surface. However, these concepts are not in competition with each other, as the main damage of articular surface usually lies on the compression side. Nevertheless, it has proven to be advantageous to take a closer look at the location of the joint impression, because some approaches allow for sufficient buttressing of a split-wedge fragment but do not provide a good exposure of central joint segments. For example, the extended posterior approach proposed by Luo allows buttressing of the medial and posterior column from the posterior border of the medial collateral ligament up to the medial border of the head of the fibula, but the joint surface that can be visualized is limited to the rim of the plateau. Combination with an extended anterolateral approach allows for reconstruction of the central articular surface (anterolaterocentral, posterolaterocentral according to Krause et al.).

#### 2.3.6. Primary total knee arthroplasty (TKA)

In elderly patients with comminuted fractures in whom anatomic reconstruction and adequate fixation are not possible, primary total knee replacement is considered a viable alternative to ORIF. Pre-existing symptomatic osteoarthritis may be another indication for primary arthroplasty (60).

The main advantage is a single-stage procedure *via* a single approach, which allows the patient to immediately weight-bear and avoids future surgery. In contrast, the development of osteoarthritis after ORIF can easily lead to a sequence of three or more procedures. Initially, the patient will require at least one operation for initial fixation of the fracture, performed through one or more “trauma approaches”. In the case of symptomatic osteoarthritis, the implants must be removed in preparation for TKA, which usually requires reopening of the scarred approaches. Only then can a secondary knee replacement be performed, which is statistically worse than primary TKA for osteoarthritis (61).

However, unlike hemiarthroplasty for femoral neck fractures, TKA for acute proximal tibial fractures is not a simple procedure. Because of instability of one or more columns, surface replacement is rarely sufficient. Revision arthroplasties are required in most cases that are candidates for primary TKA. If the collateral ligaments are involved, a hinged prosthesis should be chosen. Megaprostheses may be an option in very unstable fractures (62). Reconstruction of the anatomic joint line can be difficult, especially in fractures involving more than one column. To achieve primary load stability, zonal anchorage may sometimes require augmentations such as cones and wedges (60, 63). Accordingly, primary TKA for fractures is technically demanding and does not leave many options in the case of failure. Given a high degree of experience in revision arthroplasty, good clinical results can be achieved with this treatment strategy (60, 64, 65).

Although the literature on primary TKA for proximal tibial fractures is increasing, the evidence base is still small and the data do not lend themselves to meta-analysis. Recent systematic reviews on this topic conclude that a general recommendation for the use of primary TKA cannot be made. Nevertheless, it is considered a useful treatment option for selected patients (66, 67).

#### 2.3.7. Timing of surgery

The ideal time for definitive fracture fixation is influenced by numerous factors. Competing life-threatening injuries in polytrauma patients, concomitant neurovascular injuries, severe soft tissue damage or even the absence of a qualified team may be arguments for choosing a damage control strategy with initial application of a bridging external fixator followed by definitive reconstruction after improvement of the above-mentioned factors. While early definitive care is appropriate in low-energy-fractures (68), many authors recommend a staged protocol in high energy situations (3). However, the reported mechanism of injury alone is not a safe indicator for the choice of strategy, as complex fracture types and severe soft tissue impairment may also arise from low energy mechanisms, especially in geriatric patients with osteoporotic bone structure and neurological impairment (see Case 5, Figure 11).

#### 2.3.8. Rehabilitation

Any rehabilitation aim during the postoperative period must be adapted to each patient's individual course and depends on multiple factors: age, bone quality, type of fracture and chosen fixation method. Compliance and collateral injuries also determine the postoperative course and pace of mobilization. In terms of patient's age, elderly patients should acquire a solid osteosynthesis for early rehabilitation, as partial load of the lower extremities is often hard to achieve. Recovery was shown to be significantly slower in patients older than 40 years of age (69).

Decisions on when and how to increase the weight load to the operated tibia may depend on radiographic follow-up evaluations. Partial or no weight bearing over ten to twelve weeks is commonly recommended for tibial plateau fractures treated by CRIF, ORIF or ARIF (70), while treatment with primary arthroplasty or a circular fixator usually allows for full weight bearing (54, 60, 66, 67). However, recent studies might change treatment protocols towards less stringent weight bearing restrictions, as they show advantages of early mobilization without increasing complication rates (71).

In order to avoid consequences of long immobilization such as joint cartilage malnutrition or arthrofibrosis, passive postoperative exercise (i.e., continuous postoperative motion, CPM-unit) is necessary.

Common concepts for postoperative mobilization include limited flexion of knee joint (e.g., 0–30°) for the first two weeks with successive progression (e.g., 0 to 60° from day 15 to day 28, 0 to 90° from day 29 to day 42) and free motion thereafter.

Orthoses can protect against varus or valgus forces as well as hyperextension of the joint and limit flexion to the desired degree, which is particularly important in cases of concomitant ligamentous injuries.

Medicamentous prophylaxis of deep vein thrombosis is required until at least 20 kg of weight bearing can be allowed.

### 2.4. Complications

#### 2.4.1. Short term

Compartment syndrome following tibial plateau fracture has been described, solely as case reports, as a relatively rare complication. In a retrospective analysis of Chang and colleagues, the overall incidence of compartment syndrome was 10.3% (72). High-energy trauma (Schatzker's type IV, V, and VI) was associated with a higher incidence of compartment syndrome (30.4% in type VI).

In retrospective analyses of displaced tibial plateau fractures, treated by open reduction, infection rates from 14 to 87% were seen (73–75). Young et al. found out, that patients with postoperative infections required an average of five subsequent surgical procedures (75).

Deep vein thromboses and pulmonary embolism are severe complications after trauma. Without prophylaxis, deep vein thromboses in the lower extremities was found in 58% with adequate venographic studies (76). Additional risk factors such as age, gender, sex, vascular diseases, thromboses in the medical history or coagulation dysfunction have to be taken into consideration (77). Early start of prophylaxis is recommended (78). Both physical and medicamentous therapies with early mobilization and e.g., low molecular heparins can lower the risk of this complication.

#### 2.4.2. Long term

Using a strict definition of radiological failure of fixation, an overall rate of failure of fixation up to 31 percent was reported mostly among elderly patients (older than sixty years). According to Ali et al. loss of reduction is associated with the following factors: age over sixty years, premature weight bearing, preoperative displacement, fracture fragmentation, and severe osteoporosis (79).

The main complication after tibial plateau fracture is development of secondary osteoarthritis of the knee. It is caused by deviations from the mechanical axis and by improper reconstruction of the joint surface.

As mentioned above, articular incongruities alone seem to be relatively well tolerated in the tibial plateau (28). A major risk factor seems to be “pseudolaxity” due to reduced height of the tibial head (29).

Despite careful fracture reduction and fixation, residual symptoms are common and may range from mild aching to progressively painful post-traumatic osteoarthritis (80).

The incidence of posttraumatic osteoarthritis after tibial plateau fractures has not been firmly established, because very few long-term studies have been published (29, 81). It is reported that developing secondary osteoarthritis after tibial plateau fracture was found in 31 to 44% (82, 83). The incidence of secondary osteoarthritis was significantly higher after meniscectomy during fracture surgery (74%), than when the meniscus was preserved- either repaired or intact (82). The rate of osteoarthritis increased slightly with the age of the patients. Evidence of osteoarthritic change on follow-up radiographs after tibial plateau fracture was seen in 68% of patients aged >60 years, although only a small percentage resulted in knee replacement (84).

Although anatomical reduction may be achieved, significant joint osteoarthritis may be associated with initial articular cartilage damage (84).

Another significant variable on developing a moderate to severe grade of osteoarthritis was malalignment of mechanical leg axis of more than 5 degrees (27% of the patients) compared to patients with an anatomic knee axis (9.2%) (83). These findings strengthen the demand for correct reconstruction of axes. Therefore, it may be beneficial to correct the weight bearing axis of the leg (85). If the joint showed degenerative changes prior to trauma and if postoperative conservative treatment fails, total knee replacement is indicated. However, knee arthroplasty after tibial plateau fracture shows results inferior to primarily implanted joints due to bony destructions, deviation of axis, and insufficient ligaments (86).

Elderly patients suffering from osteoporosis and osteoarthritis at the time of tibial plateau fracture may benefit from primary total knee replacement (TKR). While the risk of complications is higher than after TKR due to primary osteoarthritis it is lower than after secondary TKR due to posttraumatic osteoarthritis (65).

Malunion or non-union of the tibial plateau can occur because of extended soft tissue trauma, iatrogenic/ traumatic harm to periosteum, infection, unstable fixation and non-addressed large bone defects. Exact preoperative planning and stable osteosynthetic fixation will reduce the incidence of these complications.

Non-union is rarely seen after low-energy plateau fractures (80) due to rich blood supply, large cross-sectional area and cancellous bone stock of the proximal tibia. This complication was observed in 4% of cases following severe fractures [predominantly type AO 41 C (87)], and their true incidence is unknown. Only few reports exist concerning treatment options in tibial plateau non-unions. In a report by Toro-Arbelaez, five intraarticular non-unions healed using standard principles of non-union reconstructive surgery, with meticulous anatomic articular reduction, rigid internal fixation, and bone grafting (88).

### 2.5. Outcome and prognostic factors

As fractures of the tibial plateau differ in a broad spectrum of fracture types, patient demographics, quality of bone stock and concomitant injuries, no uniform prognostic predictions are feasible.

#### 2.5.1. Demographic characteristics

A statistically significant association with loss of reduction was patient's age >60 years (79). Degenerative change and a mediocre functional outcome are a common occurrence following tibial plateau fractures in these patients (84). Other authors found no influence of age on the results (83).

#### 2.5.2. Functional outcome

There is significant impairment of movement and muscle function after fracture of the tibial plateau. Gaston et al. concluded, that the majority of patients have not fully recovered one year after injury. Residual flexion contractures at one year were reported and significantly slower recovery in patients older than 40 years of age (69).

Analyzing long-term functional and radiological results of surgically treated (ORIF) fractures of the tibial plateau, Rademakers and colleagues reported a mean knee range of motion (ROM) of 135 degrees after a mean period of 14 years, independent of the patient's age. Monocondylar fractures showed statistically significant better functional results compared to bicondylar fractures (83).

Long-term follow-up of high-energy fractures of tibial plateau treated with external fixation showed an average of 87% of the total ROM-arc of the contralateral knee (57).

#### 2.5.3. Patient-reported outcome measures (PROMs)

There are still few studies of proximal tibial fractures which report patient-reported outcome measures (PROMs). The Canadian Orthopaedic Trauma Society compared the functional outcome of displaced bicondylar tibial plateau fractures treated by ORIF or external fixation vs. limited open fixation. After a follow-up period of two years, there was no difference in WOMAC (Western Ontario and McMaster Universities Osteoarthritis Index) scores for pain, stiffness, or function. Regardless of treatment method, patients with this injury have significant residual limb-specific and general health deficits (73). A recent Danish cross-sectional study evaluated knee-specific and general PROMs (OKS = Oxford Knee Score, FJS-12 = Forgotten Joint Score-12, EQ-5D-5l = European Quality of Life 5 Dimensions 3 Level Version) from more than 7,000 patients with distal femur, patellar, and proximal tibia fractures. Although the authors found relatively good functional outcomes and quality of life, patients' ability to forget their knee joint after fracture was impaired (89).

## 3. Discussion

Modern management of proximal tibial fractures is built on a base of precise imaging. Conventional radiography remains useful as a simple technique providing a good overview and which can be repeated several times for follow-up. Computed tomography, however, is the diagnostic gold standard for classification and planning of fracture fixation. The steady technical improvements and increasing availability of computed tomography have led to a better understanding of the 3-dimensional nature of proximal tibial fracture, resulting in new classifications and therapeutic principles based thereon. Of particular note are the 3-column concept of Luo et al. and the 10-segment classification of Krause and coworkers (15, 17). In addition, CT can easily be supplemented by angiography, when vascular injury is suspected. MRI is a valuable complement to diagnose injuries to cartilage and ligaments.

Therapeutic options of proximal tibial fractures range from conservative treatment up to primary arthroplasty with megaprostheses. This broad spectrum might be indicative of a lack of evidence in literature, but also reflects the diversity of injury patterns in the proximal tibia. In addition, the close proximity to the complex ligamentous structures of the knee joint and the superficial position of the large neurovascular structures lead to a variety of injury constellations. Apart from the type of injury, the needs of individual patients differ markedly. The combination of factors above requires a broad portfolio of therapeutic options.

Multiply injured patients and those with severe soft tissue impairment demand a damage control strategy prioritizing life- and limb-threatening injuries. In these cases, surgery should aim at restoring the mechanical axis and achieve fracture healing while avoiding secondary damage to the impaired soft tissues. In this context, one should keep in mind that a height-stable reduction is more important than a perfect reconstruction of the articular surface (28, 29). This group of patients might also benefit from external fixation as a definitive treatment (30).

The younger and healthier the patient is, the more the surgeon should aim at a perfect reconstruction of columns and articular surface, which is probably best achieved by ORIF *via* specific approaches adapted to the respective injury (12, 19, 59). Whenever possible, minimally invasive techniques such as CRIF and ARIF should be used, alone or in combination with ORIF. Arthroscopic Reduction and Internal Fixation (ARIF) combines the advantage of visual control of the articular surface under reduction with the direct visualization and arthroscopical therapy of concomitant injuries to ligaments and cartilage (20, 32, 40, 41).

The treatment of proximal tibial fractures in elderly patients remains controversial. While it is well known that elderly patients benefit from early definitive treatment that allows them to achieve full weight bearing as soon as possible, the optimal treatment to achieve this goal is still unclear. Primary arthroplasty helps avoid fixation failure in osteoporotic bone and the disadvantages of secondary joint replacement in symptomatic osteoarthritis, but it is technically challenging and does not offer many options in case of failure (67). Proponents of primary arthroplasty emphasize the increasing risk of posttraumatic osteoarthritis with age and the advantages of single-stage surgery *via* a single approach without the risk of failure of fixation in osteoporotic bone and the problems of difficult implant removals *via* the “trauma approaches” before secondary arthroplasty can be performed. However, while radiographic signs of post-traumatic osteoarthritis after proximal tibial fracture are common, symptomatic osteoarthritis leading to total knee replacement is still a rare event. While the very low TKA conversion rate in earlier publications may be due to the limited experience with revision knee arthroplasty at that time (84), even recent studies report conversion rates below 10% at 5–10 years of follow-up (90, 91). Although risk is thought to increase with age, geriatric patients generally do well with ORIF after tibial plateau fracture (90, 92, 93). Oladeji et al. examined age-related differences after ORIF of a proximal tibial fracture and found no difference in the conversion rate to TKA. Furthermore, clinical outcomes did not differ between older patients and their younger counterparts (93). Maseda et al. reached similar conclusions in their prospective study. Comparing patients younger than and older than 65 years, they found no differences in functional outcomes, complication rates, and conversion rates for arthroplasty at 12 months (92). In addition, the above-mentioned goal of immediate weight bearing might also be realized by means of osteosynthesis without adverse effects on the functional outcome (71). However, selected patients with non-reconstructable damage to the articular surface and columns or patients with high-grade, symptomatic osteoarthritis even before fracture may benefit from primary arthroplasty (60, 64, 66).

## 4. Conclusion

The management of proximal tibial fractures is constantly evolving, affecting both diagnosis and treatment. The growing importance of CT and MRI diagnostics and thus the greater attention to concomitant injuries and posterior fracture types should be particularly emphasized. This development is also reflected in the evolution of new classifications and specific surgical approaches. At the same time, efforts are being made to restore a stepless articular surface and ligamentous and osseous stability with as little invasiveness as possible. Besides being less invasive, arthroscopic surgery allows for precise reduction of intraarticular fracture lines and treatment of concomitant injuries in the same session.
